# DL‐3‐n‐butylphthalide accelerates improvement of early‐onset post‐stroke cognitive impairment

**DOI:** 10.1002/alz70859_099003

**Published:** 2025-12-25

**Authors:** Huan Ma, Yulu Yan, Zihan Wang, Ziyu Liu, Ling Gao, Jin Wang, Qiumin Qu, Wenhui Lu

**Affiliations:** ^1^ The First Affiliated Hospital of Xi’an Jiaotong University, Xi'an, Shaanxi China; ^2^ The First Affiliated Hospital of Xi'an Jiaotong University, Xi'an, Shaanxi China

## Abstract

**Background:**

Post‐stroke cognitive impairment (PSCI) affects nearly 1/3 of stroke patients, which results in high mortality rates, high disability rates and high social and family burdens. Most PSCI occur in the early stages of stroke, known as early‐onset PSCI, for which there are no specific treatment currently. DL‐3‐n‐butylphthalide (NBP) is extracted from celery seeds and has a unique effect in protecting mitochondria and improving microcirculation. It is widely used in acute ischemic stroke patients and can significantly improve neurological damage. However, the effects of NBP on PSCI had not been determined.

**Method:**

We conducted a 24 weeks blind assess clinical trial involving persons 45 to 80 years of age with early‐onset post‐stroke cognitive impairment (PSCI). Participants were randomly assigned in a 1:1 ratio to receive NBP (200mg, 3 times a day) or conventional treatment. MOCA was used to evaluate whole cognitive function at baseline, and at 6, 12, and 24 weeks post‐treatment. Multivariate logistic regression analysis was used to evaluate the effects of NBP on the PSCI.

**Result:**

A total of 125 participants were enrolled, with 59 assigned to receive NBP and 66 to receive conventional treatment. 109 participants completed the 6‐week follow‐up. 83 participants completed the 12‐week follow‐up. 83 participants completed the 24‐week follow‐up. During 24 weeks follow‐up, the prevalence of PSCI was decrease grade after stroke in both NBP group and conventional treatment group. Prevalence of PSCI had no significant differences between the two groups at the 6‐week follow‐up and the 24‐week follow‐up. However, the prevalence of PSCI was significantly lower in the NBP group than that in conventional treatment group at the 12‐week follow‐up (52.6% vs. 75.6%, *p*=0.029). Multivariate logistic regression analysis, adjusting for age, education level, infarction subtype, and baseline MOCA score, indicated that NBP treatment was associated with a reduced risk of PSCI (OR=0.26, 95% CI 0.08‐0.81, *p*=0.020).

**Conclusion:**

Combined therapy with NBP was associated faster recovery of early‐onset PSCI. These indicated that NBP may be a potential treatment for early‐onset PSCI.

